# Nature's patchwork: How water sources and soil salinity determine the distribution and structure of halophytic plant communities in arid environments of the Eastern Pamir

**DOI:** 10.1371/journal.pone.0174496

**Published:** 2017-03-30

**Authors:** Monika Mętrak, Łukasz Chachulski, Dovutsho Navruzshoev, Paweł Pawlikowski, Elżbieta Rojan, Marcin Sulwiński, Małgorzata Suska-Malawska

**Affiliations:** 1 Faculty of Biology, Biological and Chemical Research Centre, Department of Plant Ecology and Environmental Protection, University of Warsaw, Warsaw, Poland; 2 Warsaw University of Life Sciences, Faculty of Agriculture and Botany, Department of Botany, Warsaw, Poland; 3 Kh.Yu. Yusufbekov Pamir Biological Institute of the Academy of Sciences of the Republic of Tajikistan, Khorog, Tajikistan; 4 Faculty of Geography and Regional Studies, University of Warsaw, Warsaw, Poland; Technical University in Zvolen, SLOVAKIA

## Abstract

The eastern part of the Pamir Mountains, located in Central Asia, is characterized by great climatic continentality and aridity. Wetlands developed in this hostile region are restricted to spring areas, terraces of shallow lakes or floodplains along rivers, and provide diversified ecosystem services e.g. as water reservoirs, refugia for rare species and pastures for domestic cattle. These ecosystems are particularly susceptible to climate changes, that in the Pamir Mountains result in increased temperatures, intense permafrost/glacial melt and alterations of precipitation patterns. Climatic changes affect pasture management in the mountains, causing overutilization of sites located at lower elevations. Thus, both climate and man-induced disturbances may violate the existing ecological equilibrium in high-mountain wetlands of the Eastern Pamir, posing a serious risk to their biodiversity and to food security of the local population. In this context, we sought to assess how environmental drivers (with special focus on soil features and potential water sources) shape the distribution and diversity of halophytic plant communities developed in valleys in the Eastern Pamir. This task was completed by means of a vegetation survey and comprehensive analyses of habitat conditions. The lake terraces and floodplains studied were covered by a repetitive mosaic of plant communities determined by differences in soil moisture and salinity. On lower, wetter sites, this patchwork was formed by *Blysmus rufus* dominated salt marshes, saline small sedge meadows and saline meadows with *Kobresia royleana* and *Primula pamirica*; and on drier, elevated sites, by endemic grasslands with *Hordeum brevisubulatum* and *Puccinellia* species and patches of xerohalophytic vegetation. Continuous instability of water sources and summer droughts occurring in the Pamir Mountains may lead to significant structural and functional transformations of described wetland ecosystems. Species more tolerant to decreased soil moisture and/or increased soil salinity will expand, leading to alterations of ecosystem services provided by the Pamirs’ wetlands. The described research will help to assess the current state of the wetlands and to predict directions of their future changes.

## Introduction

The hydrology of a given area, including its water supplies, ground water level and water chemistry, is a widely known factor playing a vital role in structuring and maintaining terrestrial vegetation, most notably in water-limited habitats [1–2]. According to Gilbert and Freser [3], in the case of high-mountain water bodies and wetlands, salinity may also have a comparable impact on the functioning of these ecosystems. Both hydrology and salinity of high-mountain wetlands are under a strong influence of climatic factors and their current changes, which are especially important in arid regions of the world [2, 4].

The Pamir Mountains, situated in the south-eastern part of Central Asia (mostly in Tajikistan, with outskirts reaching to Kyrgyzstan, China and Afghanistan), are characterized by extreme climatic conditions and a rather short growing season. Together with the Tibetan Plateau, the Himalayas, the Tian Shan, Karakoram, Kunlun, Hindu Kush and Hindu Raj ranges, the Pamir Mountains belong to the largest area of permafrost in mountainous regions, with the permafrost thickness estimated at less than 100 m [5]. According to the studies performed by Gorbunov at lake Karakul, the Pamirs’ permafrost is of Upper Pleistocene and Holocene origin [5].

Due to significant landscape differences, the Pamir Mountains are divided into the western and the eastern part. The Western Pamir has alpine features, with high, steep slopes (up to 7,500 m a.s.l.), deep ravines (denivelations above 4,000 m) and relatively intensive precipitation (around 1,500 mm annually, with maxima reaching 2,500 mm for the Fedchenko Meteorological Station), whereas the Eastern Pamir is an elevated plateau (3,500–4,000 m a.s.l.) surrounded by slightly lower (around 6,000 m a.s.l.), rounded peaks. This area is characterized by very low precipitation rates (between 50 and 150 mm annually), which combined with high insolation, strong winds, and average monthly air temperatures below zero from October to March, make the Eastern Pamir a cold mountainous desert with aridity index between -45 and -61 [6–13].

Due to such harsh climatic conditions, the wetlands of the Eastern Pamir, located mostly in spring areas, on terraces of brackish to saline shallow lakes or on floodplains along rivers, are totally dependent on water influxes, coming mostly from sources other than scarce precipitation (springs, permafrost meltwater, lake/river water, snow/glacial meltwater). Thus, differences in water supply and/or local weather conditions have resulted in the formation of different wetland habitats within different soil types and soil chemical properties, and consequently with totally different plant communities developed at distances of just a few meters. This repetitive mosaic of phytocoenoses has been recently described as floodplain meadows, including sedge meadows, bog sedge meadows and mountain meadows [14].

Research carried out in the Eastern Pamir to date has shown that in this semi-arid area wetlands are of paramount importance as reservoirs of meltwater from permafrost and glaciers, as resting places for migratory birds, as grazing grounds for rare herbivory species (such as Marco Polo sheep) and in general as refugia crucial for biodiversity conservation [15–17].Furthermore, they are critical for local inhabitants, who depend on fresh water sources and hence settle in the immediate vicinity of wetlands that are intensively used in livestock farming.

After the collapse of the Soviet Union, the management of pastures in the Pamir Mountains became decentralized and, together with the loss of traditional agricultural knowledge, resulted in overutilization of near-village pastures. Simultaneously, summer pastures, located at higher altitudes, became underutilized. Currently, efforts are taken to reverse this trend. In Tajikistan and Kyrgyzstan “Laws on Pastures” were passed that regulate the tenure and exploitation of pastures, and establish transboundary management practices in the Pamir Mountains. Local governments and NGOs cooperate to consolidate and optimize management practices at the level of rural municipalities, to remove barriers to the use of summer pastures (mostly improve access routes) and to enhance farmers’ knowledge of grazing methods. Yet all these activities are limited due to institutional and financial issues [13, 18–22]. Presently, different scientific, governmental and non-governmental bodies in Tajikistan are engaged in the preparation of an inventory of Pamirs’ pastures. However, they are lacking current data on ecology and biodiversity of pasturelands in the Eastern Pamir, since the most recent maps of vegetation distribution for this area were prepared in the 1950s by Stanyukovich [23]. In this context, our research fills in gaps in the existing knowledge and provides additional information on potential threats to these ecosystems.

Though unsustainable management of pastures strongly impacts high-mountain wetlands of the Eastern Pamir, the greatest threat to this ecosystems is posed by global climate changes. For the arid regions of Central Asia climate models predict a rise in temperature by 1–2°C over the next 40 years, with the greatest increase in winter [24–26]. Interestingly, precipitation models show no consistent trend for this region, probably due to a considerable natural variability of precipitation patterns in Central Asia, especially in the mountains [9, 27–30].

**Fig 1:**
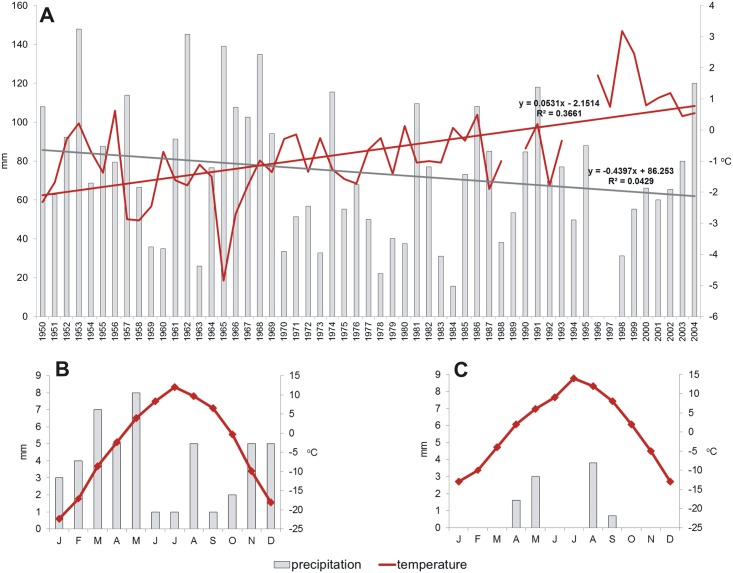
Climatic characteristics of the study area. (A) Long-term changes in average annual air temperature and total annual precipitation in Murghab (approximately 37 km from lake Rangkul), gaps are caused by the lack of data [7, 10]. Average monthly air temperatures and total monthly precipitation in 2014: (B) in Bulunkul (unpublished data from the Bulunkul meteorological station of the Tajikistan National Agency for Hydrometeorology), (C) in Murghab; lack of bars means lack of precipitation [34].

According to long-term data obtained from the meteorological station near the town of Murghab (3618 m a.s.l.) [7], over the years 1940–2005 average annual temperature had been growing by approximately 0.5°C per decade (Fig 1). This observations follow the general tendency for Tajikistan. Yet, as local climate conditions are very heterogeneous in the Pamir Mountains, there are some exceptions to this general rule—e.g. data from the meteorological station near the Bulunkul village (3767 m a.s.l.,) show a decrease in temperature by 1.1°C over the same period of time (1940–2005) [9]. According to Kayumow [10], over the years 1940–2005 an 8% increase in precipitation was observed for areas below 2500 m a.s.l., while a 3% decrease was observed for areas above 2500 m a.s.l. In the highest parts of the Eastern Pamir, the amount of precipitation was reduced even by 5–10% (Fig 1) [10].

The Pamir Mountains, as a high altitude area, are especially sensitive to climate changes, that manifest themselves in temperature increases, changes in precipitation patterns, enhanced permafrost and glacial melt and the intensification of extreme events, such as floods or landslides [27–29, 31–33]. As advancing climate change and growing human impact influence the functioning of wetland ecosystems in the Eastern Pamir, we should expect profound structural and functional transformations of wetland vegetation, mostly due to changes in water supply and soil moisture.

These transformations may strongly affect ecosystem services provided by the wetlands. For better assessment of these potential transformations, we examined the species composition and habitat requirements of the halophytic plant communities formed in the hydrogenic habitats of spring areas, floodplains and lake terraces, and determined which of the studied halophytic communities is the most tolerant to drought and salinization. Having that assessed by means of a vegetation survey combined with comprehensive habitat analyses (detailed soil survey, acquisition of meteorological data), we tried to identify the environmental factors that shape the distribution and diversity of halophytic plant communities of high-mountain wetlands, with a special focus on soil features and potential water sources; and based on statistical analyses of results obtained during our field studies.

## Materials and methods

### Site description

**Fig 2:**
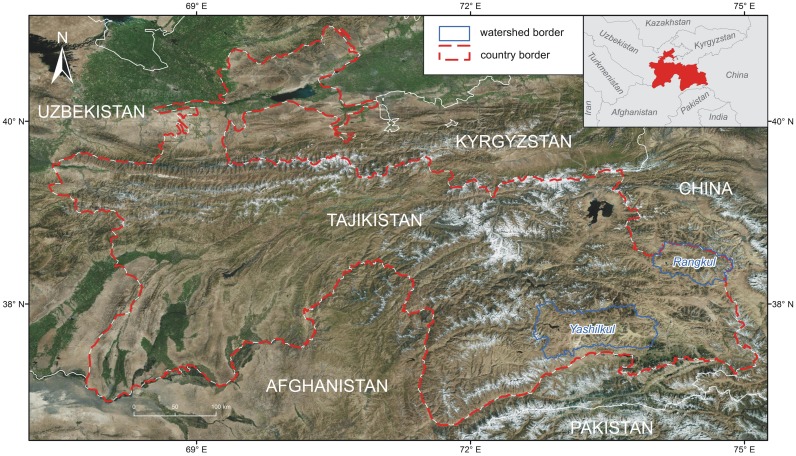
Location of watersheds in which the studied areas are located. Source: Esri, DigitalGlobe, GeoEye, Earthstar Geographics, CNES/Airbus DS, USDA, USGS, AEX, Getmapping, Aerogrid, IGN, IGP, swisstopo, and the GIS User Community.

The study areas were located in the Eastern Pamir, in the watershed of Yashilkul Lake and in the watershed of lakes Rangkul and Shorkul (Fig 2). Sampling plots were situated in the vicinity of small and medium-sized (below 10 km^2^), shallow lakes, that were surrounded by wide, flat terraces, partially covered by wetland vegetation; and on river floodplains.

Lakes Bulunkul (37°43'29"N 72°57'45"E), Sassykul (37°41'48"N 73°10'51"E) and Tuzkul (37°42'52"N 73°7'9"E) belong to the watershed of Yashilkul Lake (Fig 2) and are situated in the east–west trending valley of the river Alichur (37°43'59"N 73°7'47"E), in the climatic transition zone between the semiarid western and arid eastern parts of the Pamir Mountains. As such, this region is currently characterized by precipitation rates of approximately 50 mm per year (average for the period 2009–2014 is 59 mm, unpublished data from the Bulunkul meteorological station of the Tajikistan National Agency for Hydrometeorology) and average annual air temperature of approximately -4°C (average for the period 2009–2014 is -4.3°C, data source as above). Yet, for the period of 1951–2008 average annual precipitation was higher (86 mm) and average annual air temperature—lower (-5.4°C) [7]. Monthly averages of precipitation and temperature for the Bulunkul meteorological station in the year of our research (2014) are presented in Fig 1B.

Lake Bulunkul, located at 3767 m a.s.l., is a small lake (3.5 km^2^) supplied by the river Sulu-Tagarkaki, coming from the southern ranges. It also has a periodical inflow of waters from the much bigger Yashilkul lake. As such, Bulunkul’s waters are the least saline (up to 180 mg salts/l) and slightly alkaline (pH 8–9), in comparison to the other lakes studied. Lake Sassykul, located at 3825 m a.s.l., one of the largest (8.9 km^2^) of the lakes studied, might be supplied with water by the seasonal river Tamdy, also originating on the southern slopes. In the past it formed one basin with the small, seasonal lake Tuzkul (3798 m a.s.l., 1.8 km^2^). Now, though separated, they are both highly saline and alkaline (salinity over 100,000 mg salts/l, pH around 10).

Lake Rangkul (38°28'51"N 74°15'53"E) is located further east, at 3784 m a.s.l., in the second watershed, near the Chinese border (Fig 2). In the period of 1950–2005, this region was characterized by average precipitation of approximately 74 mm per year [7, 10]. In the year of our study, average annual precipitation was extremely low and reached only 9 mm (Fig 1C) [7]. Average annual air temperatures are milder here, than for the Yashilkul watershed (for the period 1950–2005 it is -0.7°C, and for the year of our study—0.7°C) [7, 10].There is no permanent river supplying Rangkul lake, yet it has a periodical connection with the neighboring Shorkul lake (38° 27'02"N 74° 8'92"E), located at 3780 m a.s.l. Both lakes are roughly the same size (approximately 8 km^2^), with Rangkul’s waters being slightly more alkaline and significantly less saline(pH up to 9, salinity around 400 mg/l) than Shorkul’s (pH around 8, salinity over 1200 mg/l).To the east of Rangkul lake lies a spring and seepage area, supporting the existence of wetland ecosystems.

The altitudes and areas of the lakes are cited from Tajik sources [35–36], whereas the chemical characteristics come from our own research [13].

### Vegetation survey

**Fig 3:**
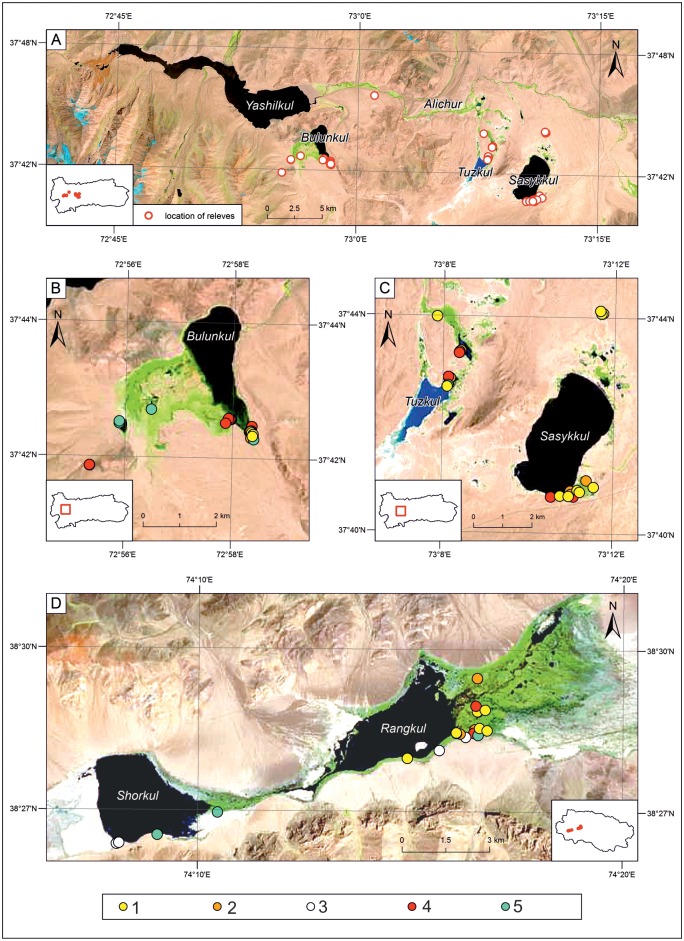
Location and classification of relevès. (A) general distribution of sampling plots in the watershed of lake Yashilkul. (B-D) Location of relevès with identified plant communities around lakes Bulunkul (B), Sassykul and Tuzkul (C), Shrokul and Rangkul (D). Source: Landsat 8 2014-06-19. Green areas denotes wetland and grassland ecosystems. 1. salt marshes dominated by *Blysmus rufus*; 2. saline small sedge meadows dominated by *Carex orbicularis* and *Carex microglochin*; 3. xerohalophytic vegetation with *Krascheninnikovia ceratoides* and *Polygonum sibiricum var*. *thomsonii*; 4. saline meadows with *Kobresia royleana* and *Primula pamirica*; 5. grasslands with *Hordeum brevisubulatum* and *Puccinellia* species.

According to the observed vegetation physiognomy and with the support of historical results from the works by Ikonnikov [37] and Stanyukovich [38], we determined the main gradient of vegetation diversity, that followed the gradient of soil moisture (from semi-desert to permanently submerged sites). Along this gradient we randomly chose 75 sampling plots (Fig 3), avoiding only extremely heterogeneous and/or extremely grazed sites (i.e. eastern part of wetlands near Rangkul lake). We used plot size determined for grasslands by Chytrý and Otýpková [39] and covering a square with side length of 3 m. From each sampling plot a relevè was obtained.

Species abundance was assessed by the Braun-Blanquet method modified according to Barkman [40]. Species were identified according to an identification key developed for the Eastern Pamir by Ikonnikov [37] and verified against specimens in the herbaria of the Experimental Station of the Academy of Sciences of the Republic of Tajikistan in Chechekty and of the Pamir Biological Institute in the city of Khorog. Species names were checked with the database of The Plants List of Kew Royal Botanic Garden [41].

For statistical analyses, we used Van der Maarel’s rank scale without any transformations [42]. The relevès from transects were classified using the modified TWINSPAN algorithm [43–44] implemented in JUICE software ([45]; pseudospecies cut levels 0, 5, 25—see [44] and [46]).

**Fig 4:**
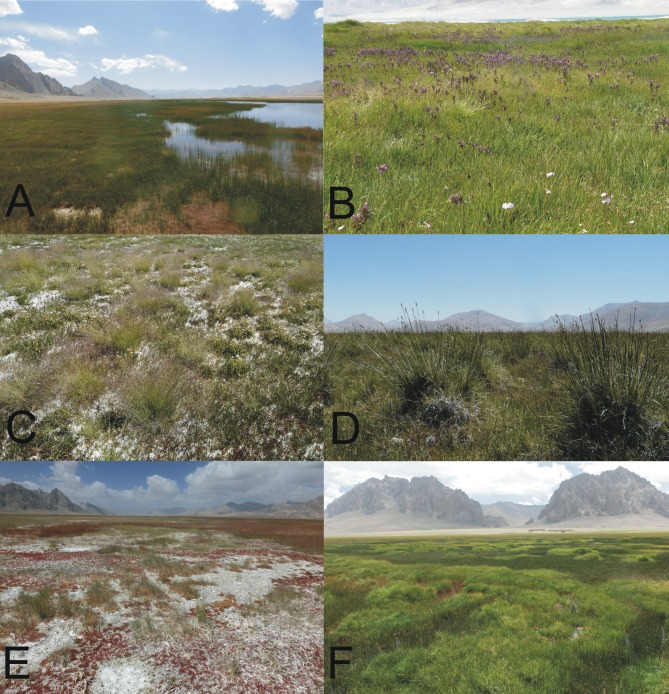
Representative examples of the described plant communities developed on floodplains and lake terraces in the Eastern Pamir. (A) salt marshes dominated by *Blysmus rufus*; (B) saline small sedge meadows dominated by *Carex orbicularis* and *Carex microglochin*; (C) grasslands with *Hordeum brevisubulatum* and *Puccinellia* species; (D) saline meadows with *Kobresia royleana* and *Primula pamirica*; (E) xerohalophytic vegetation with *Krascheninnikovia ceratoides* and *Polygonum sibiricum var*. *thomsonii*, (F) mosaic of plant communities on the terraces around Rangkul lake.

TWINSPAN classification supported the distinction of halophyte communities into: (1) salt marshes dominated by *Blysmus rufus* (Blys_ruf), (2) saline small sedge meadows dominated by *Carex orbicularis* and *Carex microglochin* (Car_om), (3) grasslands with *Hordeum brevisubulatum* and *Puccinellia* species (Pucc_Hor), (4) saline meadows with *Kobresia royleana* and *Primula pamirica* (Kob_Pri), (5) xerohalophytic vegetation with *Krascheninnikovia ceratoides* and *Polygonum sibiricum var*. *tomsonii* (Kras_Pol). Representative examples of the described communities are presented in Fig 4. For all obtained relevès, Shannon (H) and Pielou (J) indices were calculated [47].

### Soil sampling

Before the sampling, we performed a pilot investigation by preparing several soil pits and taking several cores from the studied wetlands, in a gradient of distance from water sources. In June 2014, we sampled the surface layer of soil (0–20 cm) from 55 out of the 75 plots established for relevè preparation (we were unable to sample the surface layer of soil in all the points due to equipment failure and lack of time to repeat the sampling). Soil samples were placed in paper bags, air dried and sieved before further processing (1 mm grade).

### Chemical analyses

Soil moisture was measured as the difference in mass between wet and air dried samples, expressed as percentage content of water in the dried sample. pH was measured with a Hach HQ40d device in 1 M KCl extract (1 g of soil in 10 ml of KCl solution). Electrical conductivity (EC) was measured with the Hach HQ40d device in water extract (soil: water ratio 1:5) and recalculated to electrical conductivity of soil saturated extract (ECe) at 25°C [dS/m] [48].

Concentrations of soluble cations were measured in water, and concentrations of exchangeable cations in 0.1 M BaCl_2_ extracts [49], with flame photometer Jenway PFP7 (Ca) and Flame Atomic Absorption Spectrometer Contraa700 (Na, K, Mg). Using the results obtained for exchangeable cations, we calculated:

1. the Exchangeable Sodium Percentage [%] according to the following formula: ESP = (Na^+^/CEC)x100, where ESP is Exchangeable Sodium Percentage, Na^+^ is measured exchangeable sodium expressed in cmol(+)/kg [50];
2. the sum of basic cations [cmol(+)/kg], which we decided to present below as Cation Exchange Capacity (CEC), given that concentrations of hydrogen and aluminum ions in alkaline soils are negligible [51].

Concentrations of anions were measured in water extracts, sulfates with Continuous Flow Analyzer SAN++ (methyl thymol blue method with BaCl_2_) [52]; chlorides by silver nitrate titration method; and carbonates and bicarbonates by titration method with 0.01M H_2_SO_4_[51].

As far as characterization of soil edaphic features is concerned, we analyzed total carbon and total nitrogen content (TC and TN) with a CHNS elemental analyzer NA 2500; total organic carbon content (TOC) with the same apparatus, after digestion with HCl [53]; available phosphorous by the Olsen method [51]; nitrates and ammonia ions in 0.03 M acetic acid extract [54] with Continuous Flow Analyzer SAN++.

### Statistical analyses

As the parameters studied failed to meet the assumptions of parametric tests (normal distribution and/or equal variances), non-parametric tests were used. In order to assess differences in biodiversity (expressed as species richness, Shannon and Pielou indices) among the distinguished plant communities, we used the Kruskal-Wallis test with multiple comparisons of mean ranks with Bonferroni correction. These analyses were performed on the set of 75 relevès.

Kruskal-Wallis tests with multiple comparisons of mean ranks with Bonferroni correction were also used to evaluate differences in soil features among the studied communities on the set of 55 samples taken from sampling plots.

Seeking to identify factors explaining the observed variation in soil features among the studied plant communities, we used Linear Discriminant Analyses (LDA). Changes in the frequency of the studied communities on certain types of soils (as far as salinity and total organic carbon is concerned) were assessed with Chi square tests. Analyses of soil features were performed on the data from the 55 areas established for relevès preparation.

All analyses were performed with Statistica for Windows v. 10 and with CANOCO for Windows, Version 4.5 [55].

## Results

### Biodiversity of the distinguished plant communities

The highest biodiversity, measured both by Shannon index (median H = 1.07) and by species richness (median S = 8), was recorded for saline meadows with *Kobresia royleana* and *Primula pamirica*. Apart from these two species, the highest fidelity was recorded for *Alopecurus anthoxanthoides*, *Poa angustifolia*, *Potentilla dealbata*, *Potentilla anserina* and *Gentiana leucomelaena*. Though the domination of *K*. *royleana* and grasses was strong there (median J = 0.53), due to high participation of meadow herbs, species richness was significantly higher in comparison to other communities, resulting also in higher Shannon index values (Table 1).

**Table 1:** Comparison of biodiversity indices among the distinguished plant communities. The indices were calculated for standard square sampling plots with the area of 9 m^2^. Letters in superscript denote significant differences in biodiversity indices between the distinguished plant communities.

Plant community	Species richness (S) median (min-max)	Shannon Index (H) median (min-max)	Pielou Index (J) median (min-max)
salt marshes with *B*. *rufus* (a) (N = 26)	3^d^ (2–15)	0.76 (0.14–1.58)	0.68^b^ (0.21–1.00)
sedge meadows with *C*. *orbicularis* and *C*. *microglochin* (b) (N = 19)	5^d^ (3–8)	0.66 (0.16–1.21)	0.43^ae^ (0.15–0.71)
grasslands with *Hordeum brevisubulatum* and *Puccinellia* species (c) (N = 10)	6 (2–12)	0.99 (0.14–1.71)	0.59 (0.20–0.74)
meadows with *K*. *royleana* and *P*. *pamirica* (d) (N = 13)	8^abe^ (5–11)	1.07 (0.76–1.41)	0.53 (0.36–0.65)
xerohalophytic vegetation with *K*. *ceratoides* and *P*. *sibiricum* var. *thomsonii* (e) (N = 6)	4^d^ (2–6)	0.95 (0.59–1.42)	0.73^b^ (0.61–0.88)
**Kruskal-Wallis test**	**H = 22.1333 p = 0.0002**	**H = 10.0370 p = 0.0398**	**H = 16.4112 p = 0.0025**

Both the grasslands with *Hordeum brevisubulatum* and *Puccinellia* species and xerohalophytic vegetation with *Krascheninnikovia ceratoides* and *Polygonum sibiricum*, were characterized by median Shannon index slightly lower than 1 (0.99 and 0.95, respectively); however, xerohalophytic vegetation was characterized by a more even species distribution (median J = 0.73 for xerohalophytes and median J = 0.59 for grasslands) (Table 1).

*Puccinellia* and *Hordeum* species formed a higher layer in grassland communities, while species from the genera *Glaux*, *Kochia* and *Taraxacum* constituted a lower one. These two-layered communities were dominated by halophytes, e.g. *Hordeum brevisubulatum*, *Puccinellia hackeliana*, *Puccinellia pamirica*, *Glaux maritima* and *Kochia iranica*. Similarly, species-poor xerohalophytic communities were formed mostly by halophytes, including the dominating *Krascheninnikovia ceratoides* and *Polygonum sibiricum*, and the accompanying *Carex pseudofoetida*, *Saussurea salsa* and *Leymus secalinus*.

The biodiversity of *Blysmus rufus* dominated salt marshes and saline small sedge meadows was relatively low (consequently median H = 0.76 and median H = 0.66), though in both cases diversification of species between the stands was high (Table 1). Salt marshes with the domination of *Blysmus rufus* were characterized by high fidelity of wetland species, such as *Triglochin palustre*, *Eleocharis quinqueflora*, *Carex pamirensis*, and littoral species, e.g. *Hippuris vulgaris* and *Potamogeton filiformis*. On some sites fully aquatic species were also present, such as *Potamogeton pectinatus* or *Chara canescens*, indicating stability of these particular water bodies. For small sedge meadows a strong domination of *Carex orbicularis* and *Carex microglochin* was recorded (J = 0.43, Table 1), with *Blysmus compressus*, *Triglochin maritima* and *Pedicularis rhinanthoides* as accompanying species.

A detailed synoptic table showing fidelity coefficient and percentage frequency of recorded species is presented in S1 Table.

### Site differences in soil moisture and salinity among the distinguished plant communities

**Fig 5:**
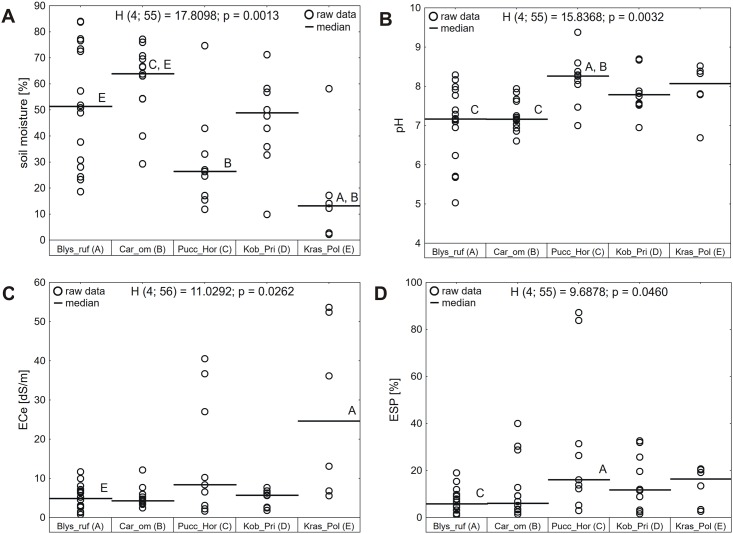
Site differences in soil moisture (A), pH (B), electrical conductivity, ECe (C), and exchangeable sodium percentage, ESP (D) among the distinguished plant communities. H—statistics of Kruskal-Wallis test, p—p value of Kruskal-Wallis test. Letters denote statistically significant (p<0.05) differences between particular plant communities. Blys_ruf: salt marshes dominated by *Blysmus rufus*; Car_om: saline small sedge meadows dominated by *Carex orbicularis* and *Carex microglochin*; Pucc_Hor: grasslands with *Hordeum brevisubulatum* and *Puccinellia* species; Kob_Pri: saline meadows with *Kobresia royleana* and *Primula pamirica*; Kras_Pol: xerohalophytic vegetation with *Krascheninnikovia ceratoides* and *Polygonum sibiricum var*. *thomsonii*.

In the studied area, halophytic plant communities developed mostly on floodplains and lake terraces on the floor of glacial valleys. Salt marshes dominated by *Blysmus rufus* formed in a wide range of habitat conditions—from dried-out areas (soil moisture less than 30%), through highly moist soils (soil moisture around 80%) to completely submerged sites, located in littoral zones of lakes and small ponds, and in temporarily water-filled hollows (stagnant water). The overall median soil moisture for *Blysmus rufus* communities was 51% (Fig 5A, detailed descriptive statistics of the studied soil parameters together with results of Kruskal-Wallis tests are presented in the S2 Table)

Similarly, saline small sedge meadows developed mainly on highly moist soils (median soil moisture 64%), yet they avoided extremities of open water and strongly dried-out areas (the lowest soil moisture recorded for small sedge beds was 29%). As such they preferred locations near springs, outflows of subsurface waters (seepage/spring zones) and small watercourses entering lakes (flowing water). Saline meadows with *Kobresia royleana* and *Primula pamirica* developed mostly on the margin of flooded areas and preferred moderately moist habitats (median soil moisture 49%). However, they also formed under drier and more moist conditions (from 10 to 70% of soil moisture).

The driest communities of grasslands with *Hordeum brevisubulatum* and *Puccinellia* species (median soil moisture 26%) and xerohalophytic vegetation with *Krascheninnikovia ceratoides* and *Polygonum sibiricum* (median soil moisture 13%) formed either at greater distance from the lakes or in elevated places, where ground water level was significantly lower, with soil surface covered by salt outcroppings (puffy solonchaks).

Soil pH was on average neutral for communities established in moist habitats and shifted to slightly alkaline as soil moisture lessened (Fig 5B). The lowest values were recorded for salt marshes dominated by *Blysmus rufus* (median value 7.16, minimum 5.03) and the highest for salt marshes with *Hordeum brevisubulatum* and *Puccinellia* species (median value 8.26, maximum 9.40), which significantly differed in pH from both *Blysmus rufus* dominated salt marshes and saline small sedge meadows.

The same pattern was visible as far as soil salinity is concerned (Fig 5C and 5D). The highest values of both electrical conductivity (ECe) and exchangeable sodium percentage (ESP) were recorded for plant communities growing on dry soils, including several hypersaline sites with ECe>16. For grasslands with *Hordeum brevisubulatum* and *Puccinellia* species, the median value of ECe was 8.35 dS/m and the median ESP was 16.02%, whereas for xerohalophytic vegetation the median ECe was 24.63 dS/m and median ESP 16.35%. Medium salinity was noticed for saline meadows with *Kobresia royleana* and *Primula pamirica* (median ECe5.68 dS/m, median ESP 11.75%), and the lowest salinity—for the most moist habitats of *Blysmus rufus* salt marshes (median ECe 4.85 dS/m, median ESP 5.80%) and saline small sedge meadows (median ECe 4.22 dS/m, median ESP 6.04%).

**Fig 6:**
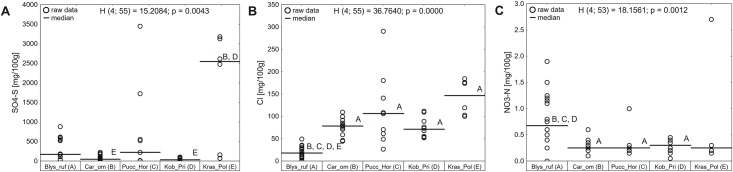
Site differences in content of sulfates (A), content of chlorides (B) and content of nitrates (C) among the distinguished plant communities. H—statistics of Kruskal-Wallis test, p—p value of Kruskal-Wallis test. Letters denote statistically significant (p<0.05) differences between particular plant communities. Blys_ruf: salt marshes dominated by *Blysmus rufus*; Car_om: saline small sedge meadows dominated by *Carex orbicularis* and *Carex microglochin*; Pucc_Hor: grasslands with *Hordeum brevisubulatum* and *Puccinellia* species; Kob_Pri: saline meadows with *Kobresia royleana* and *Primula pamirica*; Kras_Pol: xerohalophytic vegetation with *Krascheninnikovia ceratoides* and *Polygonum sibiricum var*. *thomsonii*.

The content of chloride ions in soils (Fig 6B) was related to their moisture and salinity—with the chlorides content being the lowest in moist soils from *Blysmus rufus* dominated salt marshes (median value 17.85 mg Cl^-^/100g), drastically growing in soils from saline small sedge meadows (median value 78.22 mg Cl^-^/100g) and saline meadows with *Kobresia royleana* and *Primula pamirica* (median value 71.05 mg Cl^-^/100g) to reach the maximum in soils from grasslands with *Hordeum brevisubulatum* and *Puccinellia* species and from xerohalophytic vegetation (median values consequently 106.40 and 146.65 mg Cl^-^/100g).

Neither sulfates nor nitrates showed such a clear pattern (Fig 6A and 6C). In the case of sulfates, the highest content was observed in xerohalophytic communities (median content 2546 mg SO_4_^2-^-S /100g), which were established in a very wide range of sulfates content (from 69 to 3181 mg SO_4_^2-^-S /100g), followed by grasslands with *Hordeum brevisubulatum* and *Puccinellia* species (median content 222.07 mg SO_4_^2-^-S /100g) and salt marshes dominated by *Blysmus rufus* (median content 172 mg SO_4_^2-^-S /100g). The lowest sulfates content was recorded in soils from saline small sedge meadows (median content 46 mg SO_4_^2-^-S /100g) and saline meadows with *Kobresia royleana* and *Primula pamirica* (median content 31 mg SO_4_^2-^-S /100g). In the case of nitrates, the highest values were recorded in soils from *Blysmus rufus* dominated salt marshes (median content 0.67 mg NO_3_^-^-N/ 100g), the remaining communities were characterized by nitrates content ranging from 0.25 to 0.30 mg NO_3_^-^-N/ 100g.

According to a Chi square test, the studied plant communities showed no preferences for soils with specific salinity, defined according to the US Salinity Laboratory Staff Classification [56]. (Chi^2^ = 17.1923; df = 12; p = 0.1425).

### Site differences in soil organic matter content among the distinguished plant communities

For the purpose of our study, we distinguished three main soil types on which the studied plant communities were formed, in agreement with the available literature [57–58] (Table 2).

**Table 2:** Differences between observed and expected values in a Chi square test of plant communities’ occurrence on soils characterized by different content of organic matter. TOC—total organic carbon.

Plant community	Soil type and characteristics		
	high mountain alluvial peat soils (HM APS)	high mountain alluvial meadow soils (HM AMS)	high mountain loamy alluvial soils (HM LAS)
	organic, TOC>12%, HISTIC horizon	organic, 6%<TOC<12%, MELANIC horizon	mineral, formed on alluvial deposits
salt marshes with *B*. *rufus*	3.64	-3.49	-0.14
sedge meadows with *C*. *orbicularis* and *C*. *microglochin*	0.18	4.94	-5.13
grasslands with *Hordeum brevisubulatum* and *Puccinellia* species	-1.45	-1.96	3.42
meadows with *K*. *royleana* and *P*. *pamirica*	-1.73	1.82	-0.09
xerohalophytes with *K*. *ceratoides* and *P*. *sibiricum* var. *thomsonii*	-0.64	-1.31	1.94
**Chi square statistics**	**29.0099; df = 8; p = 0.0003**		

According to a Chi square test, presented in Table 2, salt marshes dominated by *Blysmus rufus* showed clear preferences for high mountain alluvial peat soils (HM APS), while being extremely rare on high mountain alluvial meadow soils (HM AMS), which were in turn preferred by saline small sedge meadows. Excluded from HM AMS, *Blysmus rufus* communities formed stable phytocoenoses on high mountain loamy alluvial soils (HM LAS), while saline small sedge meadows apparently avoided such habitats. Saline meadows with *Kobresia royleana* and *Primula pamirica* formed mostly on HM AMS, whereas grasslands with *Hordeum brevisubulatum* and *Puccinellia* species, together with xerohalophytic communities with *Krascheninnikovia ceratoides* and *Polygonum sibiricum*, developed on HM LAS. Grasslands with *Hordeum brevisubulatum* and *Puccinellia* species were very frequent on this type of soil.

In terms of soil organic matter, salt marshes dominated by *Blysmus rufus*, saline small sedge meadows and saline meadows with *Kobresia royleana* and *Primula pamirica* were characterized by higher values of total nitrogen, total carbon and total organic carbon than grasslands with *Hordeum brevisubulatum* and *Puccinellia* species, and xerohalophytic vegetation. Yet, the observed differences were at a rather low level of statistical significance (p values in Kruskal-Wallis tests between 0.0460 and 0.0726). The opposite trend was recorded for C/N ratio, with higher values for dry communities and lower for *Blysmus rufus* dominated salt marshes and both types of meadows (p value in Kruskal-Wallis test 0.0524).

More significant differences were recorded in the content of nitrates (described earlier), the content of ammonia ions (p value in Kruskal-Wallis tests 0.0333) and the content of available phosphates (p value in Kruskal-Wallis tests 0.0126). Apart from xerohalophytic vegetation, all communities were characterized by comparable amounts of ammonia ions (4.90–15.38 mg NH_4_^+^-N/100 g of soil) and phosphates (0.37–6.70 mg PO_4_^3-^-P/100 g of soil). Soils from xerohalophytic communities contained significantly lower amounts of ammonia ions (5.29–9.51 mg NH_4_^+^-N/100 g of soil) and phosphates (0.25–1.46 mg PO_4_^3-^-P/100 g of soil).

### Site differences summarized with multi-dimensional analysis

**Fig 7:**
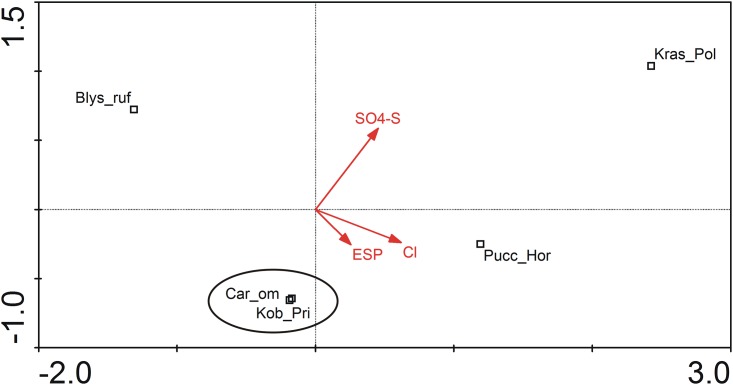
Linear discriminant analysis of the studied communities with 15 environmental parameters as independent variables (λ_1_ = 0.677, λ_2_ = 0.664, λ_3_ = 0.477, λ_4_ = 0,187). For the purpose of clarity, only parameters with significant impact on observed variation between the studied communities were included in the graph. ESP–Exchangeable Sodium Percentage. Blys_ruf: salt marshes dominated by *Blysmus rufus*; Car_om: saline small sedge meadows dominated by *Carex orbicularis* and *Carex microglochin*; Pucc_Hor: grasslands with *Hordeum brevisubulatum* and *Puccinellia* species; Kob_Pri: saline meadows with *Kobresia royleana* and *Primula pamirica*; Kras_Pol: xerohalophytic vegetation with *Krascheninnikovia ceratoides* and *Polygonum sibiricum var*. *thomsonii*.

According to a Linear Discriminant Analysis (LDA, Fig 7) performed on the whole dataset (55 samples from 5 plant communities, 15 environmental variables), only three parameters had significant influence on the variation observed between the studied communities. Changes in chloride content in soil explained 13.9% of the observed variation (p = 0.0001), changes in sulfate content in soil explain 8.4% (p = 0.0007) and changes in exchangeable sodium percentage (ESP) explained 3.5% of the variation observed between the studied communities (p = 0.0484).

Xerohalophytic vegetation (Knor_Eur) and grasslands with *Hordeum brevisubulatum* and *Puccinellia* species (Pucc_Hor) were characterized by relatively high values of the above-mentioned parameters, with sulfate salinization characteristic for soils from xerohalophytic communities and chloride salinization characteristic for soils from grasslands with *Hordeum brevisubulatum* and *Puccinellia* species.

*Blysmus rufus* dominated salt marshes (Blys_ruf) were significantly less saline. Both saline small sedge meadows (Car_om) and saline meadows with *Kobresia royleana* and *Primula pamirica* (Kob_Pri) showed great similarity in required habitat conditions. They preferred sites poor in sulfates, with a rather low content of chlorides, yet relatively rich in sodium ions.

## Discussion

The history of botanical studies in the Pamir Mountains is quite long, with the first descriptions of the Pamir’s vegetation being prepared at the beginning of the twentieth century by Olga Fedchenko. Yet, it was not until 1963 that a complete botanical description of both the eastern and western parts of the mountains was issued by Ikonnikov (with a short review of vegetation types included) [37], who based his comprehensive publication on the works of other Russian botanists, mainly Stanyukovich [38], Ovczinnikov [59–60] and Stieshenko [61–62]. Ikonnikov’s flora was updated by Mikhailova in the 1970s [63], and his research was continued by Agakhanjanz, who after the collapse of the Soviet Union started collaborating with German botanists [14, 64]. Recently, it is mostly German and Polish botanists who have been working in the Pamir Mountains. Their studies are dedicated either to chosen plant taxonomical and/or ecological groups (e.g. [65–67]), or to the impact of grazing on meadow and wetland ecosystems (e.g.[14, 18–20]). Although digitalized materials from Agakhanjanz’s studies have recently been published [14], there are still no sufficient ecological analyses of the Eastern Pamir, with the most recent maps of vegetation distribution having been prepared in the 1950s by Stanyukovich [23]. Hence, our research will update and complement the existing knowledge on the Eastern Pamir vegetation, with a focus on its wetlands.

The zonation of plant communities on the slopes of the investigated valleys was similar and comparable to the results of earlier investigations in the Pamir Mountains [68].Due to their specific water regime, the wetlands of Central Asian mountains are dominated by halophytic species, able to successfully complete their life cycle in saline habitats [63, 69–70]. There are several classifications of halophytes based either on ecological or physiological characteristics of plant species, or combining them both [70–74]. Regardless of which classification is used, we should remember that due to differences in climate and vegetation, in distinct areas of the world prevail halophytes form different classes [75]. Hence, we should use a classification that describes local halophyte diversity in the best possible way. In the case of the cold deserts of the Pamir Mountains, we adopted the classification proposed by Khan [70–71], who based it on his own field research and a thorough review of literature dedicated mostly to the cold deserts of Central Asia.

According to the literature, the wetlands in this region are dominated by hydrohalophytes from the Cyperaceae and Poaceae families, with xerohalophytes from the Chenopodiaceae family present on drier sites [70–71]. Our survey demonstrated that wetland vegetation in the Eastern Pamir share some floristic similarities with halophytic communities known from temperate and Mediterranean climatic zones of Europe (e.g. [74–79]), assigned (according to various approaches) to the classes Juncetea maritimi (or Asteretea tripolium) and Festuco-Puccinellietea (or Puccinellio-Salicornietea). The similarities mentioned include the dominance of grasses from the genus *Puccinellia*, as well as species like *Blysmus rufus*, *Glaux maritima* and *Triglochin maritima*. Such communities often develop in supralittoral zones of saline water bodies [80]. The most numerous group of halophytic species identified in our study area were hydrohalophytes e.g. *Blysmus rufus*, *Triglochin palustre* and *Triglochin maritima* or *Carex orbicularis*. A less abundant group was comprised of xerohalophytes typical for saline steppes and deserts, such as *Kochia iranica*, *Glaux maritima or Krascheninnikovia ceratoides*. Furthermore, some psammohalophyte species were also recorded e.g. *Polygonum sibiricum* and *Leymus dasystachys*. The participation of different halophytic species in the distinguished plant communities reflected differences in soil moisture and salinity. Hydrohalophytes dominated among the diagnostic species of the most moist and least saline salt marshes (including inundated species) and small sedge meadows, while the diagnostic species of highly saline xerohalophytic vegetation belonged to either xero- or psammohalophytes. The diagnostic species of moderately moist and saline grasslands and *Kobresia* meadows belonged to both hydro- and xerohalophytes.

As far as species composition is concerned, we identified 15 species of halophytes listed for the northern mountains of Pakistan by Khan [70–71] and 4 species of halophytes listed for Badakhshan (Tajikistan) by Ikonnikov [37]. Comparing our vegetation clusters with vegetation classes reported from Europe [76], we found several resemblances. The most diverse communities of saline meadows with *Kobresia royleana* and *Primula pamirica* share a few species with the Molinio-Arrhenatheretea class, as semi-natural flooded meadows. According to traditional pasture management practices, these communities are used as winter pastures, hence they are located at relatively low altitudes and are rich in species of meadow herbs. As such, they are less exploited during summer, which allows the occurring species to complete their reproductive cycles, and supports relatively high biodiversity of these ecosystems [13, 81–82]. However, the current situation in the Pamir Mountains is far from this ideal pattern of seasonal movement of herds between winter and summer pastures (vertical transhumance). Nowadays herders tend to over-utilize near village winter pastures, since traditional agricultural knowledge was partially lost as a result of collectivization during the Soviet times. Moreover, high altitude summer pastures are often poorly accessible due to the condition of roads and bridges [13, 21, 28]. Therefore, all pastures located in the Pamirs’ valleys are already grazed in summer. For the purpose of our research, we chose less grazed sites in order to properly assess the taxonomic diversity of plant communities. Hence, we are aware that man-induced transformations in species composition and vegetation structure might be better pronounced on more degraded sites.

In comparison to saline meadows with *Kobresia royleana* and *Primula pamirica*, communities of both wetter and drier habitats were less diversified, either due to strong domination of particular species (salt marshes dominated by *Blysmus rufus*, saline small sedge meadows dominated by *Carex orbicularis* and *Carex microglochin*) or because of extreme conditions (xerohalophyte communities). Inundated or not, *Blysmus* communities (that often remained in spatial relation to small sedge meadows and aquatic vegetation with *Hippuris vulgaris* and *Potamogeton* spp.), because of the dominant species, show resemblance to maritime salt-marshes of the *Juncetea maritimi* (*Asteretea tripolii*) class, while drier grasslands with *Puccinellia* and *Hordeum brevisubulatu*m, bear affinities with the *Festuco-Pucinellietea* (= *Puccinelio-Salicornietea*) class of continental salt-marshes and salt-steppes. Interestingly, the later vegetation type was characterized by relatively low numbers of highly specialized halophyte species and, due to the dominance of halophyte species from the *Puccinellia* genus that have ranges limited to the Pamir Mountains (e.g. *Puccinellia pamirica*, *Puccinellia hackeliana*), can be considered endemic. Small sedge meadows dominated by *Carex orbicularis* and *Carex microglochin*, bound to spring (less saline) habitats, share a number of species with fen vegetation of the *Scheuchzerio-Caricetea nigrae* class.

Applying the classification scheme proposed by Golub [83] for halophytic vegetation in the former USSR and Mongolia, the vegetation we studied could be placed within the *Halerpestetalia* order, which embraces Mongolian moderately saline meadows on permafrost. Species composition similarities between Mongolian and Pamir halophytic vegetation are particularly apparent in the case of 3 (out of 5) vegetation clusters distinguished in the present study: sedge meadows with *Carex orbicularis* and Carex *microglochin*, grasslands with *Hordeum brevisubulatum* and *Puccinellia* species and xerohalophytic vegetation with *Krascheninnikovia ceratoides* and *Polygonum sibiricum*. The floristic affinities can be easily explained using both similar habitat conditions (permafrost, moderately saline soils) and geographic proximity. However, it should be pointed out that Golub’s [83] analysis does not cover the mountains of Central Asia, hence clarifying the exact syntaxonomical position of the vegetation studied needs extensive, pan-Euro-Asiatic comparative analysis.

As for halophytes, the most important factors determining species distribution are soil moisture and salinity [84–85]; the abundance and diversity of species observed in the Pamir Mountains were controlled by the availability of water, which in turn was a result of topographic features, both in meso- and micro-scale. The soils on which the studied communities were established were formed on Quaternary sediments, mostly on silty and/or loamy alluvial deposits and on peats that developed in post-glacial endorheic hollows with remaining permafrost. Wetlands were supplied by springs and/or seepages (soligenous wetlands), meltwater from discontinuous permafrost located at a depth of 70 cm in the areas surrounding Bulunkul and Rangkul lakes (own unpublished data) or by saline lake water (topogenous wetlands). Soils formed under such conditions are subjected to cryogenic processes (including frost stirring), resulting in the development of frost mounds [86], that enhance the patchwork character of the studied area. Due to the summer decrease of ground water level, organic soils mineralize and a moorsh layer is formed. If this process is followed by an increase in salinity, it will lead to the formation of marshy/meadow solonchaks [6, 58]. On the margins of wetlands and on elevated sites in the vicinity of lakes, xerosols developed, characterized by a calcic horizon and usually occupied by grasslands with *Hordeum brevisubulatu*m and *Puccinellia* species, or characterized by a gypsic horizon and occupied by xerohalopytic vegetation with *Krascheninnikovia ceratoides* and *Polygonum sibiricum var*. *thomsonii*.

Though evolutionary adaptations enable halophytes to survive under conditions of high and fluctuating soil salinity and allow them to outcompete generalist species, they remain susceptible to high concentrations of NaCl during germination [84, 87–89, 90–92]. In our case, the factors significantly influencing the distribution of the studied plant communities included exchangeable sodium percentage, suggesting the importance of the proportion of sodium to other cations, and as far as anions are concerned, chloride and sulfate concentrations. In regard to soil salinity, periodical changes in water supplies (precipitation, permafrost melt, flooding) seem to be critical for the stability and biodiversity of halophyte communities. Seasonal influxes of water leach salts deeper into the soil horizons. Thus they make the soil surface less saline, which allows germination and consolidation of halophyte seedlings. These processes are the most visible in hollows, where water stays longer, dissolving and leaching more salts. Hence, in highly differentiated terrain, such as our study area, it is hollows that are covered by more lush and productive vegetation. However, dry periods are also important for the development of halophyte communities. Due to the high evaporation rate, salts accumulate and precipitate in and on the surface layer of the soil. Increased salinity prevents colonization by glycophyte species. Moreover, salt stress positively influences the germination of halophyte seeds in the next moist period (osmopriming) [89–90, 93–94].

This fragile equilibrium can be easily violated by advancing climate changes [10, 26–27, 29, 30–31]. Currently in the Pamir Mountains, intense snow and glacial melt alternates river patterns, resulting in increased water runoff in spring and its strong reduction in summer [13, 28, 95]. Unstable water flow, combined with increased spring precipitation, leads to the flooding of wetland vegetation in April/May and subsequent drought in summer [28, 95]. Villages at lower elevations (2,000–3,000 m a.s.l.) are already reporting loss of valuable agricultural land to high water levels, while herders declare that fodder on high altitude summer pastures is often dried-out and animals are not gaining the necessary weight to sustain them through the winter [28]. With a gradual increase of average monthly temperatures, active permafrost layer is melting more intensively during summer, changing habitat conditions accordingly to meso- and microtopography of a given area [86, 96]. Intense permafrost melting in early summer results in short-term rise in soil moisture, followed by a late summer decrease of ground water level. These changes lead to the intensification of organic matter decomposition, that results in an increased availability of nutrients and alterations in biogeochemical cycles of some elements [86, 96].

Thus, continuous instability of water resources and summer droughts may lead to the desiccation of high altitude wetlands and to the replacement of hydrohalophyte communities by species more tolerant to water deficits [97]. Hence, we can expect the expansion of salt marshes with obligatory halophytic species and xerohalophytic vegetation in the Pamirs’ valleys. Moreover, an extended growing season (according to [28] 15–20 days longer than 15 years ago) in the Pamir Mountains may promote the growth of more generalist plant species at higher altitudes, inducing further structural and functional alternations of halophytic communities on lake terraces and flood plains. Such changes can be devastating for the wetlands, especially in the Eastern Pamir, where their role as refugia and biodiversity hotspots (including endemic species) is exceptionally important [15–17].

## Conclusions

The distribution of plant communities in the valleys of the Eastern Pamir is determined by the water supplies of a given area, comprised of peripheral and underwater springs supplying lakes, meltwater from permafrost remaining relatively close to soil surface, ephemeral run-off from mountain slopes and sparse precipitation. Flat terraces around lakes Bulunkul, Sassykul, Tuzkul, Rangkul and Shorkul, and floodplains along the Alichur river are covered by a repetitive mosaic of distinct plant communities, formed on lower, wetter sites by *Blysmus rufus* dominated salt marshes, saline small sedge meadows and saline meadows with *Kobresia royleana* and *Primula pamirica*, and on drier, elevated sites by grasslands with *Hordeum brevisubulatum* and *Puccinellia* species and patches of xerohalophytic vegetation. Interestingly, grassland communities can be considered endemic, as they are dominated by halophyte species from the *Puccinellia* genus that have ranges limited to the Pamir Mountains (e.g. *Puccinellia pamirica*, *Puccinellia hackeliana*).

Since pasturelands are rare in the Pamir Mountains, both saline meadows with *Carex orbicularis* and *Carex microglochin*, and with *Kobresia royleana* and *Primula pamirica* are of paramount importance to the local population, as grazing sites for yaks and sheep. Yet, continuous instability of water sources and summer droughts, both resulting from global climate changes, may lead to significant structural and functional transformations of these wetland ecosystems. Species more tolerant to decreased soil moisture and/or increased soil salinity will expand, leading to alterations of ecosystem services provided by wetlands. Generally, the area of available pastures may lessen and fodder quality may decrease, mostly due to a higher participation of obligatory halophytes and xerohalophytic species. As a result, grazing pressure on the existing pasturelands may increase, further altering their species composition and diversity. Thus, both climate and man-induced disturbances may violate the existing ecological equilibrium in high-mountain wetlands of the Eastern Pamir, posing a serious risk to their biodiversity and to the food security of the local population.

## Supporting information

S1 Table Synoptic table with modified fidelity phi coefficient and percentage frequency (upper index). Only species with phi coefficient above 10 were included.
(DOCX)

S2 Table Descriptive statistics of soils from the studied plant communities. ECe—electrical conductivity, ESP—Exchangeable Sodium Percentage, CEC—Cation Exchange Capacity, TOC—Total Organic Carbon, Pav—available phosphorous (Olsen method).
(XLSX)
