# Supplementary material for: Nature's patchwork: How water sources and soil salinity determine the distribution and structure of halophytic plant communities in arid environments of the Eastern Pamir
Source: PLoS One. 2017 Mar 30;12(3):e0174496. doi: 10.1371/journal.pone.0174496 (PMC5373547; doi:10.1371/journal.pone.0174496)
Supplement: S1 Table — Only species with phi coefficient above 10 were included. (DOCX) [file pone.0174496.s001.docx]

**S1 Table. Synoptic table with modified fidelity phi coefficient and percentage frequency (upper index).** Only species with phi coefficient above 10 were included.

| **Plant community** | **salt marshes with *B. rufus*** | **sedge meadows with *C. orbicularis* and *C. microglochin*** | **grasslands with *Hordeum* *brevisubulatum* and *Puccinelia* species** | **meadows with *K. royleana* and *P. pamirica*** | **xerohalophytic vegetation with *K. ceratoides* and *P. sibiricum* var. *thomsonii*** |
| --- | --- | --- | --- | --- | --- |
| **No. of relevés** | 26 | 19 | 11 | 13 | 6 |
| *Blysmus rufus* | 91.8^100^ | --- ^5^ | --- ^9^ | --- ^.^ | --- ^.^ |
| *Potamogeton filiformis* | 57.7 ^44^ | --- ^5^ | --- ^.^ | --- ^.^ | --- ^.^ |
| *Triglochin palustre* | 38.3 ^70^ | --- ^32^ | 12.0 ^45^ | --- ^23^ | --- ^.^ |
| *Eleocharis quinqueflora* | 29.8 ^19^ | --- ^.^ | --- ^.^ | 5.5 ^8^ | --- ^.^ |
| *Carex orbicularis* | --- ^19^ | 60.1 ^95^ | --- ^9^ | 25.7 ^62^ | --- ^.^ |
| *Carex microglochin* | --- ^26^ | 42.5 ^68^ | --- ^.^ | 26.5 ^54^ | --- ^.^ |
| *Blysmus compressus* | --- ^.^ | 42.2 ^53^ | 21.6 ^36^ | --- ^8^ | --- ^.^ |
| *Triglochin maritima* | 14.2 ^19^ | 35.9 ^32^ | --- ^.^ | --- ^.^ | --- ^.^ |
| *Pedicularis rhinanthoides* | --- ^7^ | 33.3 ^32^ | --- ^.^ | 7.2 ^15^ | --- ^.^ |
| *Glaux maritima* | --- ^4^ | --- ^5^ | 50.1 ^45^ | --- ^8^ | --- ^.^ |
| *Hordeum brevisubulatum* | --- ^.^ | --- ^.^ | 46.2 ^45^ | 13.6 ^23^ | --- ^.^ |
| *Taraxacum brevirostre* | --- ^.^ | --- ^.^ | 42.0 ^45^ | 21.6 ^31^ | --- ^.^ |
| *Puccinellia hackeliana* | --- ^.^ | --- ^.^ | 38.9 ^18^ | --- ^.^ | --- ^.^ |
| *Taraxacum bessarabicum* | --- ^.^ | --- ^.^ | 38.9 ^18^ | --- ^.^ | --- ^.^ |
| *Kochia iranica* | --- ^.^ | --- ^5^ | 36.6 ^36^ | --- ^.^ | 11.7 ^20^ |
| *Puccinellia pamirica* | --- ^26^ | --- ^16^ | 27.2 ^73^ | 8.2 ^54^ | 14.4 ^60^ |
| *Kobresia schoenoides* | --- ^4^ | --- ^5^ | 28.1 ^18^ | --- ^.^ | --- ^.^ |
| *Gentiana leucomelaena* | --- ^.^ | --- ^5^ | --- ^9^ | 58.6 ^54^ | --- ^.^ |
| *Calamagrostis anthoxanthoides* | --- ^.^ | --- ^.^ | --- ^.^ | 57.7 ^38^ | --- ^.^ |
| *Kobresia royleana* | --- ^.^ | --- ^16^ | 5.4 ^36^ | 57.4 ^85^ | --- ^20^ |
| *Potentilla anserina* | --- ^7^ | --- ^.^ | 3.1 ^18^ | 51.9 ^54^ | --- ^.^ |
| *Primula pamirica* | --- ^19^ | 15.2 ^58^ | 2.6 ^45^ | 50.0 ^92^ | --- ^.^ |
| *Poa angustifolia* | --- ^.^ | --- ^.^ | --- ^.^ | 44.0 ^23^ | --- ^.^ |
| *Polygonum sibiricum var. thomsonii* | --- ^4^ | --- ^5^ | --- ^18^ | --- ^8^ | 82.3^100^ |
| *Krascheninnikovia ceratoides* | --- ^.^ | --- ^.^ | --- ^.^ | --- ^.^ | 59.0 ^40^ |
| *Carex pseudofoetida* | --- ^15^ | --- ^5^ | --- ^.^ | --- ^15^ | 52.0 ^60^ |
| *Saussurea salsa* | --- ^.^ | --- ^.^ | --- ^.^ | --- ^.^ | 40.8 ^20^ |
| *Leymus dasystachys* | --- ^.^ | --- ^.^ | 7.0 ^9^ | --- ^.^ | 30.3 ^20^ |
| *Potentilla dealbata* | --- ^.^ | --- ^.^ | 18.0 ^18^ | 26.9 ^23^ | --- ^.^ |
| *Gentiana karelinii* | --- ^.^ | --- ^.^ | --- ^.^ | 25.0 ^8^ | --- ^.^ |
| *Carex pycnostachya* | --- ^.^ | --- ^.^ | --- ^.^ | 25.0 ^8^ | --- ^.^ |
| *Calamagrostis turkestanica* | ‚ --- ^.^ | --- ^.^ | --- ^.^ | 25.0 ^8^ | --- ^.^ |
| *Plantago gentianoides* | ‚ --- ^.^ | --- ^.^ | 9.7 ^9^ | 24.3 ^15^ | --- ^.^ |
| *Cerastium cerastoides* | --- ^.^ | 8.4 ^5^ | --- ^.^ | 16.1 ^8^ | --- ^.^ |
| *Saxifraga hirculus* | 1.0 ^4^ | 5.4 ^5^ | --- ^.^ | 12.1 ^8^ | --- ^.^ |
| *Stellaria brachypetala* | 1.0 ^4^ | 5.4 ^5^ | --- ^.^ | 12.1 ^8^ | --- ^.^ |
| *Calamagrostis stricta* | 1.0 ^4^ | 5.4 ^5^ | --- ^.^ | 12.1 ^8^ | --- ^.^ |
| *Astragalus kuschakevitschii* | --- ^.^ | --- ^.^ | 15.9 ^9^ | 12.0 ^8^ | --- ^.^ |
| *Taraxacum leucanthum* | --- ^4^ | --- ^.^ | --- ^9^ | 9.7 ^15^ | 17.6 ^20^ |
| *Alopecurus mucronatus* | --- ^.^ | 18.8 ^16^ | 5.2 ^9^ | 2.4 ^8^ | --- ^.^ |
| *Carex pamirensis* | 17.3 ^4^ | --- ^.^ | --- ^.^ | --- ^.^ | --- ^.^ |
| *Chara canescens* | 17.3 ^4^ | --- ^.^ | --- ^.^ | --- ^.^ | --- ^.^ |
| *Hippuris vulgaris* | 17.3 ^4^ | --- ^.^ | --- ^.^ | --- ^.^ | --- ^.^ |
| *Pedicularis cheilanthifolia* | 17.3 ^4^ | --- ^.^ | --- ^.^ | --- ^.^ | --- ^.^ |
| *Potamogeton pectinatus* | 17.3 ^4^ | --- ^.^ | --- ^.^ | --- ^.^ | --- ^.^ |
| *Puccinellia distans* | --- ^.^ | 20.6 ^5^ | --- ^.^ | --- ^.^ | --- ^.^ |
| *Myriophyllum spicatum* | --- ^.^ | 20.6 ^5^ | --- ^.^ | --- ^.^ | --- ^.^ |
| *Catabrosa aquatica* | 7.2 ^4^ | 13.1 ^5^ | --- ^.^ | --- ^.^ | --- ^.^ |
| *Festuca sulcata* | --- ^.^ | --- ^.^ | 27.2 ^9^ | --- ^.^ | --- ^.^ |
| *Carex stenophylla subsp. stenophylloides* | --- ^.^ | --- ^.^ | 27.2 ^9^ | --- ^.^ | --- ^.^ |
| *Utricularia sp.* | 15.5 ^7^ | 8.7 ^5^ | --- ^.^ | --- ^.^ | --- ^.^ |
| *Ranunculus natans* | --- ^4^ | 4.4 ^5^ | 14.7 ^9^ | --- ^.^ | --- ^.^ |
